# Engineered Optogenetic Circuits In Yeast with Self‐Sustained Outputs

**DOI:** 10.1002/advs.75865

**Published:** 2026-05-27

**Authors:** Cong Fan, Haofeng Chen, Yan Wang, Junyi Wang, Yueyang Chen, Yang Zhang, Jinting Guan, Jifeng Yuan

**Affiliations:** ^1^ State Key Laboratory of Cellular Stress Biology School of Life Sciences Faculty of Medicine and Life Sciences Xiamen University Fujian China; ^2^ Department of Automation Xiamen University Fujian China; ^3^ School of Advanced Interdisciplinary Biomedical Sciences Faculty of Medicine and Life Sciences Xiamen University Fujian China

**Keywords:** genetic circuits, optogenetics, quorum‐sensing, surrogate messenger, synthetic biology

## Abstract

Optoswitches are of particular interest to the metabolic engineering community, as light has a superior advantage of tunability and reversibility. However, the light‐shading effect at industrial scales remains an unsolved challenge. Here, we report optogenetic quorum‐sensing (OptoQS) circuits to induce and maintain a sustained gene expression at the population level by transient light stimulation. In particular, we reprogram the pheromone‐responsive G‐protein coupled receptor (GPCR) signaling cascade in *Saccharomyces cerevisiae* to effectively record transient light inputs. Once the transient light input is recorded as a form of α‐factor accumulation, the surrogate messenger can diffuse and transmit the signal across the cell population. Eventually, we successfully demonstrated the utility of the OptoQS circuit for metabolic regulation of 3‐hydroxypropionate biosynthesis. Based on the promising results from OptoQS circuits, we envision that the flexibility of our design might be explored for the future fabrication of various genetic circuits to record other transient physical stimuli.

## Graphical abstract

Optogenetic quorum‐sensing (OptoQS) circuit enables *Saccharomyces cerevisiae* to record transient light stimulus with self‐sustained outputs. Panel **a** indicates the design of the OptoQS circuit based on G‐protein coupled receptor (GPCR) signaling cascade. Panel **b** indicates the mode of action of surrogate messenger‐mediated signal transmission at the cell population level.

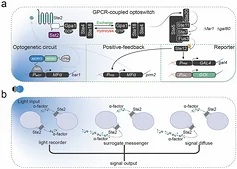

## 1 Introduction

During the microbial synthesis of biochemicals, the imbalance of metabolic flux distribution inevitably creates conflicts between cellular growth and biochemical production [1, 2]. Metabolic engineers prefer to employ inducible promoters to separate the growth phase and the production phase. However, conventional inducible systems necessitate the additives like isopropyl‐β‐D‐thiogalactopyranoside (IPTG), arabinose, rhamnose, or tetracycline [3, 4, 5], which pose considerable cost concerns, particularly in large‐scale industrial operations [6]. With the advancement of synthetic biology and metabolic engineering, there is a shifted trend toward fabricating inducer‐free expression systems, such as autonomous quorum‐sensing and physical stimuli‐regulated biochemical production processes. For instance, a pathway‐independent quorum‐sensing circuit was developed to autonomously switch gene expression based on the cell density [7]. By reprogramming the pheromone‐responsive G‐protein coupled receptor (GPCR) signaling pathway in *Saccharomyces cerevisiae*, the engineered cell–cell communication system enables dynamic regulation of metabolic pathways [8, 9, 10]. Nevertheless, the precise timing and adjusting of quorum‐sensing‐regulated gene expressions still require extensive trial‐and‐error studies for the optimal strain performance.

As light‐switchable genetic controls offer exceptional reversibility and spatiotemporal tunability over chemical inducers, there is a growing interest in developing optoswitches for metabolic engineering applications. Light‐switchable transcription modules have been implemented for non‐toxic, tunable gene expressions [11, 12, 13, 14]. Recently, Zhao et al. reported that optogenetic control based on EL222 from *Erythrobacter litoralis* could be used to dynamically regulate the cellular metabolism for improved chemical productions, and the engineered yeast produced 8.49 g/L of isobutanol and 2.38 g/L of 2‐methyl‐1‐butanol by periodic blue light pulses to tune the enzyme levels during the fed‐batch fermentation process [15]. To further reduce the light exposure, a blue light‐activated circuit with signal amplification was designed for high‐level gene expression in *S. cerevisiae* [16]. Under industrial‐scale conditions or solid phase fermentation, the penetration of light across the extremely dense culture is relatively challenging [17]. To mitigate the light‐shading effect, Zhao et al. proposed the use of a signal amplification module by coupling to the galactose‐regulated (GAL) system in yeast for a more rapid and sustained gene expression [18]. Recently, similar layered genetic designs have been successfully implemented in various microorganisms for fabricating complex genetic circuits with improved circuit performances such as amplified and sustained signal outputs [16, 19, 20, 21, 22, 23]. Although active stirring during the fermentation could improve the light accessibility, it still results in cell‐to‐cell expression heterogeneity due to uneven light exposures.

To address the light‐shading effect, we propose a framework of optogenetic quorum‐sensing (OptoQS) circuits to allow the yeast population to sense and record transient light stimuli, thereby inducing and maintaining a sustained gene expression at the population level by a short period of light stimulation. In brief, the system combines light‐sensing transcriptional modules to capture the light input and transduce to the synthesis of diffusible quorum‐sensing molecules. Transient light inputs first initiate the synthesis of diffusible quorum‐sensing molecules, whereas the quorum‐sensing molecules can further induce the genes‐of‐interest (GOI) expressions under the quorum‐sensing responsive circuits. Once quorum‐sensing molecules accumulate to a threshold level, they trigger a sustained GOI expression without the need for further light exposure. OptoQS circuits are therefore expected to address the light‐shading effect encountered during the high‐density or solid‐phase fermentation processes.

**FIGURE 1:**
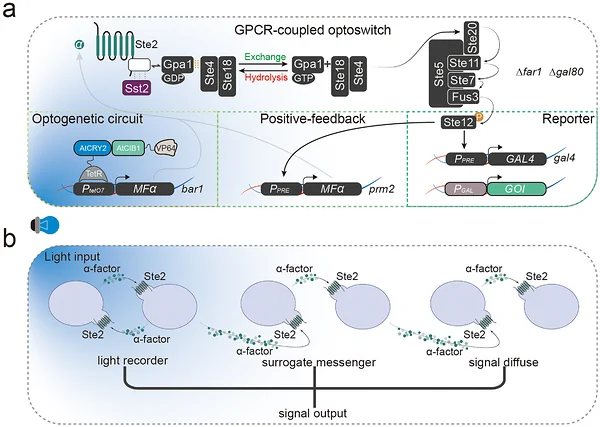
Design of optogenetic quorum‐sensing (OptoQS) circuit in *S. cerevisiae* to record a transient light stimulus. (a) Optogenetic control of GPCR‐mediated quorum‐sensing circuit. The cryptochromes (CRY2/CIB1) from *A. thaliana* are used to sense the blue light (450 nm). The blue light triggers the heterodimerization of VP64‐CIB1 and TetR‐CRY2, which brings together the two‐component transcriptional activator complex to give an induced expression of α‐factor under the control of *P_tetO7_* promoter. Subsequently, the GPCR signaling cascade activates the Gal4 activator under the control of the pheromone‐responsive (PRE) promoter. Eventually, gene‐of‐interest (GOI) under the control of galactose‐regulated promoters are triggered for expression. (b) The mode of action of surrogate messenger mediated signal transmission at the cell population level. Once the transient light input is recorded as a form of α‐factor accumulation, the surrogate messenger of α‐factor can diffuse and transmit the signal to the neighboring cell population.

In this study, we attempted to harness the yeast GPCR signal cascade with α‐factor as the diffusible quorum‐sensing molecule to record the transient light inputs (Figure 1a). In particular, the exposure of light to the yeast cells can be first recorded in the form of α‐factor accumulation, which continuously activates the mating pheromone GPCR‐mediated signaling pathway to drive the GOI expressions at the cell population level (Figure 1b). As a proof‐of‐concept, we successfully developed an OptoQS circuit based on the GPCR signaling cascade in *S. cerevisiae* and the light‐switchable transcriptional module of CRY2/CIB1 cryptochromes from *Arabidopsis thaliana* [13, 24], and the engineered OptoQS system could effectively record transient light inputs with tunable gene expression outputs. Lastly, we showcased the applicability of our OptoQS circuits for effective regulation of the metabolic pathway for 3‐hydroxypropionate (3HP) production. Taken together, we envision the feature of the OptoQS system to record transient light inputs with tunability will eventually address the light‐shading effect under industrial‐scale fermentations.

## 2 Results

### 2.1 Engineering the GPCR Dynamics by Coupling to the Yeast Galactose Regulon

**FIGURE 2:**
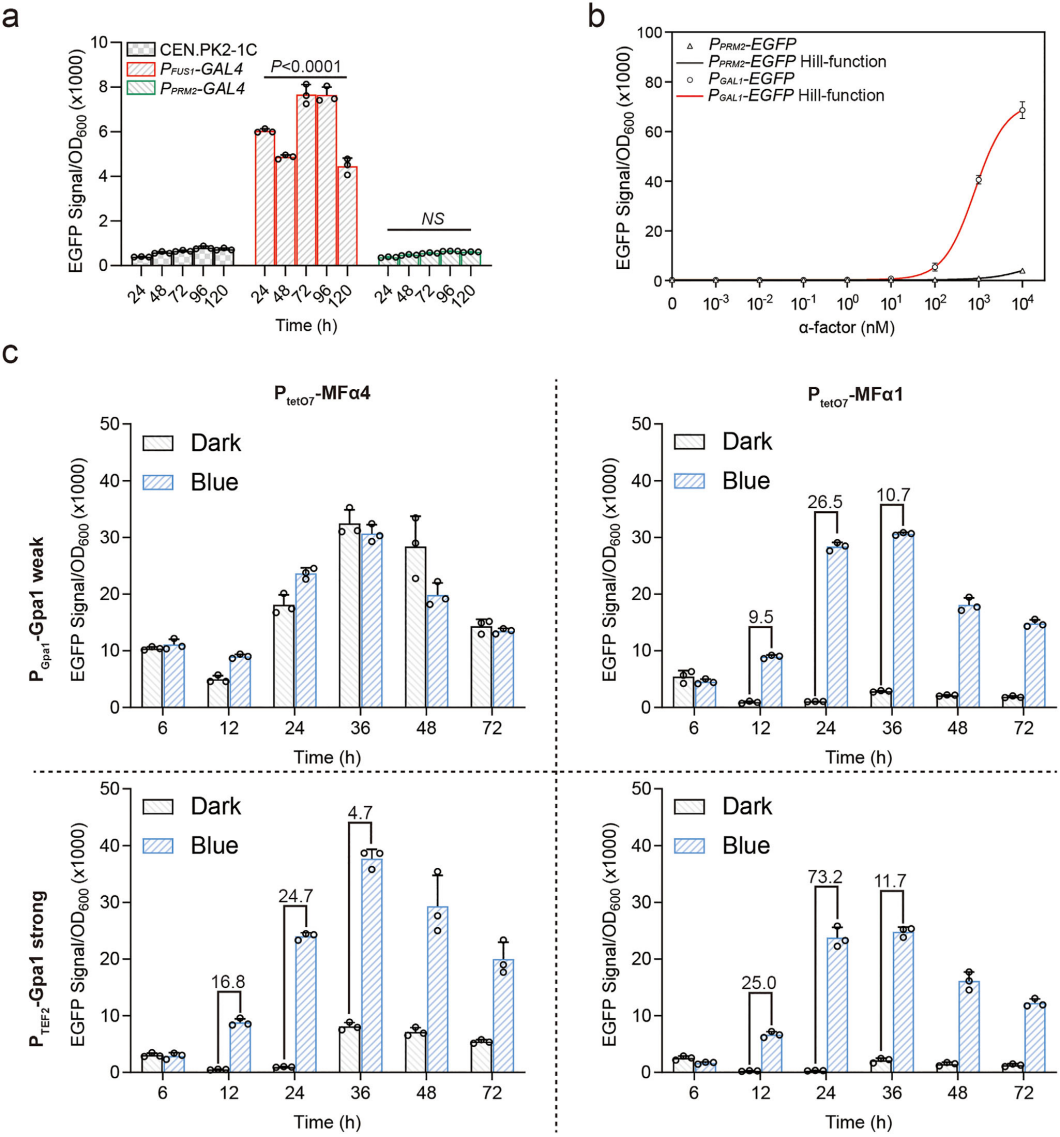
Engineering the yeast galactose regulon to sense a transient light stimulus through the GPCR signaling pathway. (a) Comparison of the tightness of *P_FUS1_‐GAL4* and *P_PRM2_‐GAL4* using a reporter gene. Plasmid pRS425 with *P_GAL1_‐EGFP* is used as a downstream fluorescent reporting module. CEN.PK2‐1C harboring pRS425 plasmid with *P_GAL1_‐EGFP* serves as a control. All strains are cultivated in synthetic complete (SC) media with glucose as the sole carbon source. (b) Dose‐response curves of genetic circuit treated with different concentrations of α‐factor. Strain ScG2 harboring either pRS425 plasmid with *P_GAL1_‐EGFP* or *P_PRM2_‐EGFP* were compared under the same condition using glucose‐containing SC medium. (c) The effect of α‐factor copies and Gpa1 overexpression on the performance of genetic circuits. All experiments were conducted as following: after pre‐cultivated in YPD media at 30°C for 6 h in the dark, the engineered strains were exposed to 100 µmol·m^−^^2^·s^−^^1^ blue light for 6 h and returned to dark cultivation. Data were obtained from three biologically independent samples and are presented as mean ± standard deviation. One‐way ANOVA, followed by Bonferroni's multiple comparisons test with 95% confidence intervals was performed using GraphPad Prism 9.0.1 software and *p*‐values are indicated in the graph. NS denotes not significant (*p* value > 0.05).

To improve the magnitude of signal outputs from the GPCR signaling pathway, we decided to control the galactose‐regulated (GAL) system via the pheromone‐responsive regulation of the Gal4 activator (Figure 1a), as the GAL system can be induced over 1000‐fold on the addition of galactose [25]. Upon disrupting *Gal80*‐mediated transcriptional repression, the refactored GPCR genetic circuit would allow the GAL regulon to function in the glucose‐containing medium. Since *P_FUS1_* provides a stable transcriptional induction in response to pheromone signaling [26], it serves as an appropriate promoter for driving sensitive downstream activation. We first used the *P_FUS1_* promoter to control the Gal4 expression in a parental yeast strain with *∆gal80 ∆far1*, whereas the *FAR1* gene was deleted to prevent α‐factor‐triggered G1 arrest [27]. Consistent with previous reports [26, 28], a relatively leaky expression of the *EGFP* gene was observed in the engineered strain ScG1 (*∆far1 ∆gal80 P_FUS1_‐Gal4*) transformed with a plasmid expressing enhanced green fluorescent protein (EGFP) under the *GAL1* promoter, which might be caused by the high basal level of *P_FUS1_* (Figure 2a). As *P_PRM2_* requires the joint regulation by Ste12 and Kar4, which makes the response of *P_PRM2_* to the mating pheromone in a more stringent manner [29, 30], we then replaced the leaky *P_FUS1_* with *P_PRM2_*, and the resulting strain ScG2 (*∆far1 ∆gal80 P_PRM2_‐GAL4*) gave no noticeable leaky expression of EGFP when compared to that of the control (Figure 2a).

As shown in Figure 2b, a clear dose‐dependent EGFP expression in response to different concentrations of externally supplemented α‐factor was observed in strain ScG2 harboring pRS425Gal1‐EGFP, suggesting that the input signal of α‐factor was transduced to *P_GAL1_*‐*EGFP*. To confirm that coupling the GPCR signaling pathway to the GAL system would improve the signal magnitude, we also investigated the dose‐response curve in the engineered strain ScG2 with *P_PRM2_*‐*EGFP*. In comparison, the control group with pRS425Prm2‐EGFP only produced a very low amount of EGFP, further confirming that the signal amplification mediated by *P_PRM2_*‐*GAL4* was more effective in enhancing the magnitude of signal output than the conventional GOI expression using direct pheromone‐responsive promoters. Noteworthily, due to the stringent regulation of *P_PRM2_*‐*GAL4*, strain ScG2 with *P_GAL1_*‐*EGFP* had a half‐maximal effective concentration (EC50) of ∼813 nm α‐factor and a Hill slope of 1.40 (Figure S1).

### 2.2 Recording Transient Light Stimuli via the GPCR Signaling Pathway

To further explore the GPCR signaling pathway to record transient light stimuli, we explored *A. thaliana* CRY2/CIB1 cryptochromes [13, 24] to sense blue light stimuli and convert the light signal into the form of α‐factor accumulation. The blue light (450 nm) triggers the heterodimerization of TetR‐CRY2 and VP64‐CIB1, which activates the expression of α‐factor under TetR‐responsive promoters [31]. We incorporated the light switchable transcription modules in strain ScG2 (ScBase: *∆far1 ∆gal80 P_PRM2_‐GAL4 ∆aro10::VP64‐CIB1 ∆adh6::TetR‐CRY2*). Subsequently, the *P_tetO7_‐MFα4* module (four copies of α‐factor in the natural tandem way [32]) was integrated at the *BAR1* locus to abolish the active degradation of α‐factor [33], and the resulting strain was designated as ScOptoQS(α)1 (a ScBase derivative with *∆bar1::P_tetO7_‐MFα4*). Surprisingly, there was no obvious difference in EGFP expression after the blue light treatment (Figure 2c). Considering the noise level of genetic circuits can be adjusted by manipulating the promoter strengths and key components involved in the GPCR signal transduction [34], we next attempted to tune the α‐factor copy number to address the leaky issue in our OptoQS circuit. As shown in Figure 2c, more than a 26.5‐fold ON/OFF ratio of EGFP expression in strain ScOptoQS(α)2 (a ScBase derivative with *∆bar1::P_tetO7_‐MFα1*) was obtained at 24 h when the yeast cells were treated with 6 h blue light.

As the basal activity of the GPCR signaling pathway can also be reduced by overexpression of Gα (encoded by the *GPA1* gene) [35], we further investigated the effect of Gpa1 overexpression on the performance of our OptoQS circuit. As shown in Figure 2c, we noticed that the Gpa1 overexpression could also mitigate the leaky issue of ScOptoQS(α)1, and the ON/OFF ratio reached 24.7 in strain ScOptoQS(α)3 (a ScOptoQS(α)1 derivative with *P_TEF2_‐GPA1*). Moreover, we found that combining the Gpa1 overexpression with MFα1 could enable strain ScOptoQS(α)4 with a better ON/OFF ratio, and a 73.2‐fold change of EGFP expression at 24 h was achieved upon the blue light exposure. We also confirmed that the EGFP expression in strain ScOptoQS(α)4 was dependent on light durations, and tunable gene expressions under different light exposures were observed (Figure S2). However, the maximum EGFP output in strain ScOptoQS(α)4 was slightly reduced when compared to that of strain ScOptoQS(α)2, indicating that the Gα not only acts as a “noise reducer” to lower the basal activity of the response, but also leads to a reduction in the maximal signal output by acting as a “signal blocker” [34]. As the supernatant from ScOptoQS(α)4 cultures had a sufficient amount of α factor in triggering the EGFP expression in strain ScG2 (Figure S3), we reasoned that the reduced EGFP after 48 h in strain ScOptoQS(α)4 was potentially caused by the exhaustion of nutrition for EGFP synthesis, and potential desensitization of the GPCR signal pathway.

### 2.3 A Feedforward Autoinduction Loop to Improve the OptoQS Circuit

**FIGURE 3:**
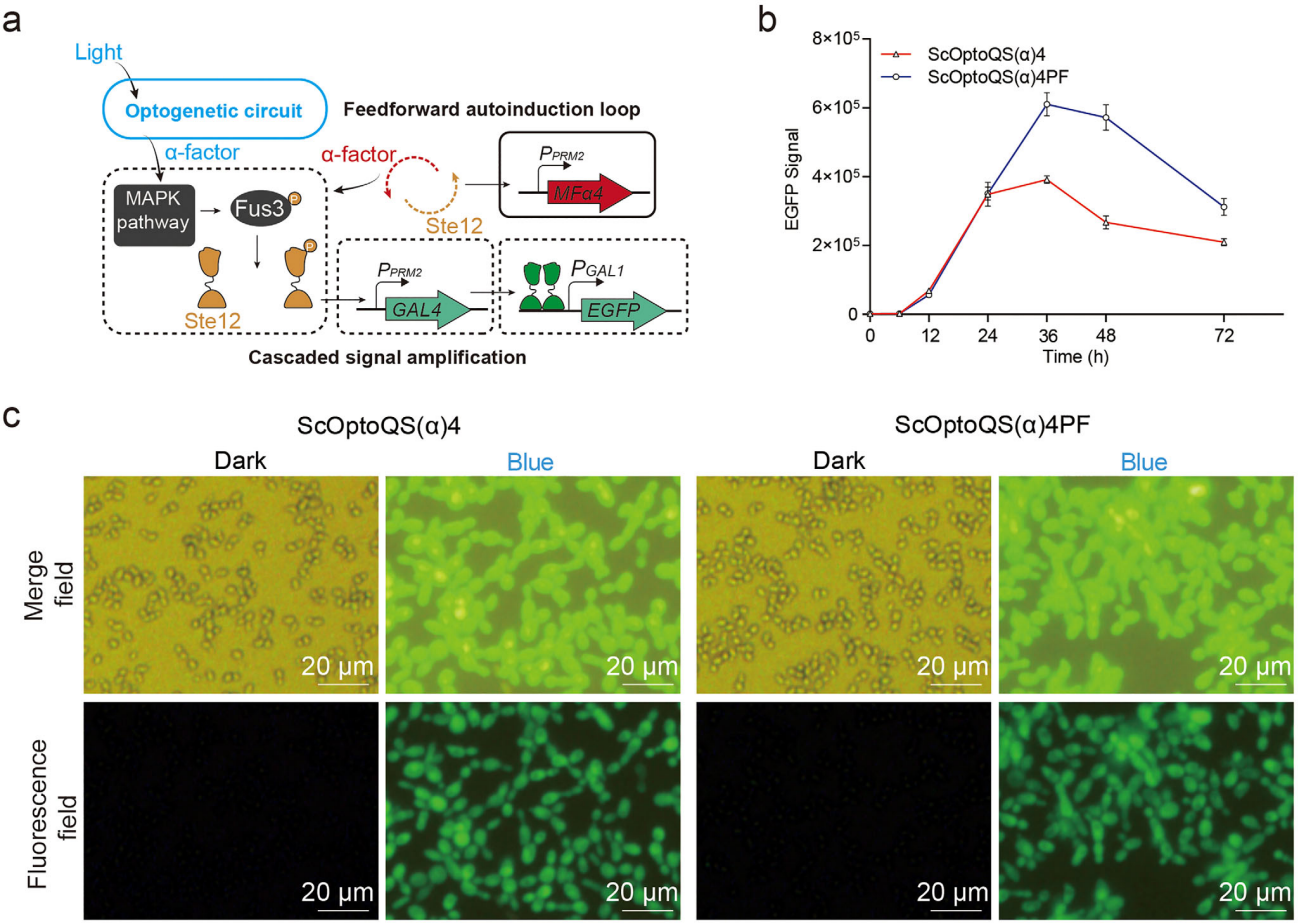
Engineering a feedforward autoinduction loop of α‐factor synthesis to boost the circuit performance. (a) Schematic diagram of a feedforward autoinduction loop mediated by *P_PRM2_‐MFα4*‐deriven α‐factor synthesis. The loop enables pheromone‐responsive self‐amplification of α‐factor production to enhance circuit outputs. (b) The effect of a feedforward autoinduction loop mediated by *P_PRM2_‐MFα4* synthesis on the circuit performance. Strain ScOptoQS(α)4PF was a derivative of ScOptoQS(α)4 with *P_PRM2_‐MFα4*. Both strain ScOptoQS(α)4 and ScOptoQS(α)4PF have chromosomal integration of *∆gal7‐10‐1::P_GAL1_*‐*EGFP*. **c**. Representative fluorescence microscopy images of EGFP expression at the cell population level in strains ScOptoQS(α)4 and ScOptoQS(α)4PF. All experiments were carried out as following: after pre‐cultivated in YPD media at 30°C for 6 h in the dark, the engineered strains were exposed to 100 µmol·m^−^^2^·s^−^^1^ blue light for 6 h and returned to dark cultivation. Data were obtained from three biologically independent samples and are presented as mean ± standard deviation.

To better transduce the light signal across the cell population and to enhance the magnitude of signal outputs, we aimed to introduce a feedforward autoinduction loop to control α‐factor levels by implementing pheromone‐responsive synthesis of α‐factor (Figure 3a). As shown in Figure 3b, strain ScOptoQS(α)4PF (a ScOptoQS(α)4 derivative with *P_PRM2_*‐*MFα4*) with the feedforward autoinduction loop could substantially enhance the signal output over that of ScOptoQS(α)4 by more than 1.5‐fold at 36 h. Transient optogenetic activation induces the production of a diffusible signal that reinforces the signaling loop, thereby sustaining GAL‐driven expression after light removal. This network‐level persistence of gene expression activity is mechanistically distinct from DNA‐encoded storage memory, whereby transient light input is converted into sustained population‐wide activation through signal propagation. However, as the Kar4‐mediated activation of the *P_PRM2_* promoter requires a relatively high concentration of α‐factor [30], no obvious change in EGFP expression in the early 24 h was observed (Figure 3b). Since pheromone‐responsive *cis*‐elements can alter the responses of synthetic promoters to pheromones [36], future rational promoter engineering by introducing multiple regulation elements would be possible to improve the performance of synthetic genetic circuits. Fluorescence microscopy confirmed that the EGFP expression in strain ScOptoQS(α)4 and ScOptoQS(α)4PF was activated at the cell population level (Figure 3c).

**FIGURE 4:**
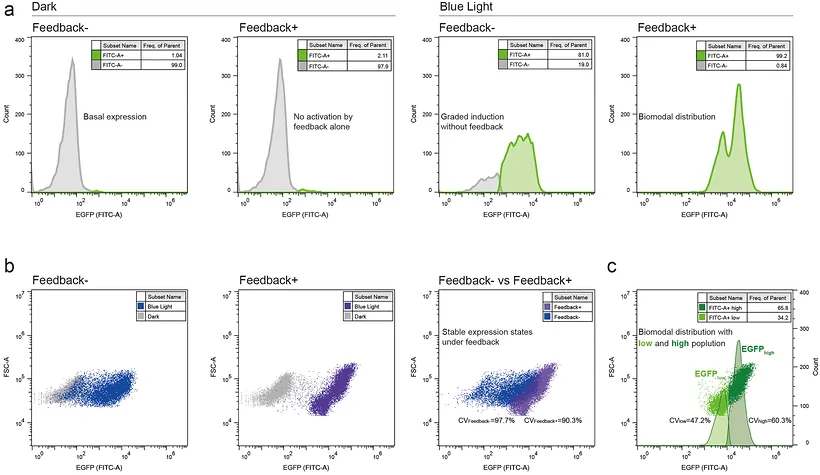
Flow cytometry analysis of population‐level signal propagation and single‐cell variability. (a) Representative flow cytometry histograms showing the GFP expression states under light with positive feedback. (b) Flow cytometry scatter plots showing the single‐cell variability of α‐factor‐mediated different ScOptoQS strains. (c) Flow cytometry results showing two well‐separated low and high GFP expression populations. All experiments were carried out as following: after pre‐cultivated in YPD media at 30°C for 6 h in the dark, the engineered strains were exposed to 100 µmol·m^−^^2^·s^−^^1^ blue light for 6 h and returned to dark cultivation. Representative images were presented from three biological triplicates with reproducible results.

To further confirm the population‐level signal propagation and answer the single‐cell variability, we further applied flow cytometry analysis. In the absence of feedback, blue light stimulation led to a unimodal distribution of GFP expression, indicating a graded response across the cell population (Figure 4a). In contrast, cells harboring the positive feedback circuit exhibited a clearly bimodal distribution under blue light, consisting of two well‐separated populations corresponding to low and high GFP expression states (Figure 4a,c). Importantly, both populations remained GFP‐positive, suggesting that positive feedback does not simply switch gene expression ON or OFF, but rather discretizes expression into distinct activation states. To determine whether distinct expression states are associated with phenotypic differences, we analyzed cell size using forward scatter (FSC) measurements. Upon blue light stimulation, cells lacking feedback showed only a modest increase in FSC, consistent with a graded response (Figure 4b). In contrast, cells with positive feedback displayed a distinct subpopulation with elevated FSC under blue light (Figure 4b), indicating the emergence of a larger‐size cell phenotype. To directly assess the relationship between gene expression and cell size, we performed joint analysis of GFP intensity and FSC. This revealed two clearly separated clusters corresponding to low‐expression/small cells and high‐expression/large cells (Figure 4c).

Furthermore, the coefficient of variation (CV) analysis showed that variability slightly decreased under blue light in the presence of positive feedback. This reduction in variability, together with the bimodal distribution, suggests that cells are partitioned into more stable expression states rather than exhibiting increased stochastic noise. As shown in Figure 4a,b, we also observed that there was heterogeneity in EGFP signal intensity across the cell population. This heterogeneity can be interpreted within the framework of “secrete‐and‐sense” circuits [37]. In such systems, cells simultaneously secrete and sense the same signaling molecule, enabling two modes of communication: self‐communication (autocrine‐like signaling) and neighbor‐communication (paracrine or quorum‐like signaling). The balance between these two modes determines the overall population behavior. This variation may arise from the design of our engineered strain, in which each cell simultaneously functions as both a signal sender and a signal receiver. Cells that effectively capture their own secreted α‐factor might experience stronger local signaling (self‐communication), leading to higher activation states. In contrast, cells that primarily respond to extracellular α‐factor originating from neighboring cells exhibit comparatively lower activation levels. The difference between self‐sensing and neighbor‐sensing may result in a non‐uniform EGFP output across the population, which might be addressed in the future by tuning the secretion rate and the receptor abundance [37].

Next, we applied the ordinary differential equations (ODEs) to simulate the reaction process of the entire system to explore the accumulation level of signal molecules under instantaneous light stimulation. As can be found in Figure S4a, the experimental results for EGFP expressions are in good agreement with the modeling results of the positive feedback system under 6 h of light activation. In addition, to further verify the recording ability of the positive feedback system, we simulated the levels of signal molecules after 6 h of light activation to show their accumulation (Figure S4b). The model prediction shows that the concentration of α‐factor signal molecules increases with time with the feedforward autoinduction loop. Therefore, the model further suggested that the positive feedback system could respond to light stimulation and had an improved recording function, supporting the conclusion that transient light input can be effectively propagated and maintained at the population level through the engineered circuit dynamics.

### 2.4 Implementation of the OptoQS Circuit for Metabolic Engineering Applications

**FIGURE 5:**
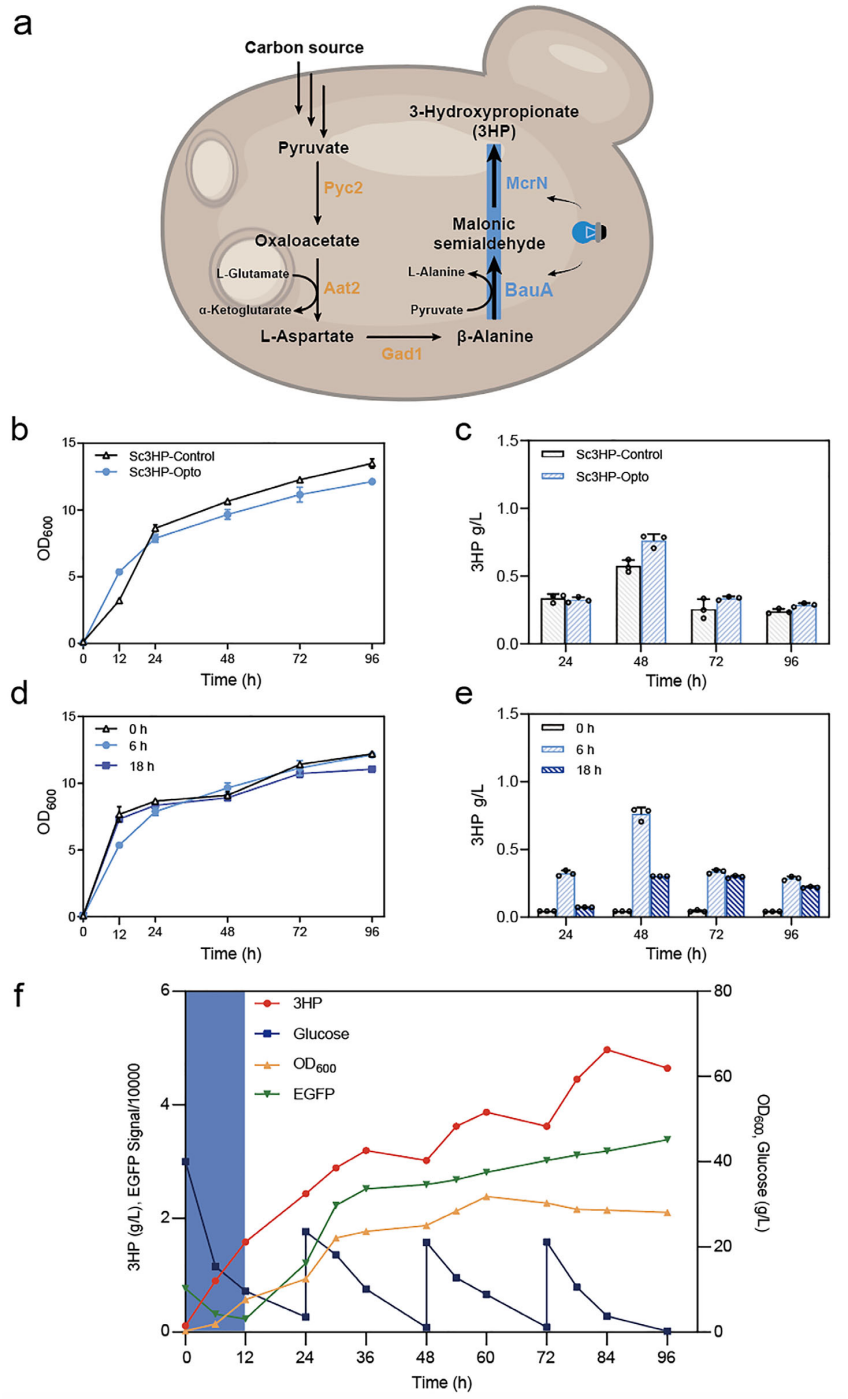
The OptoQS circuit for effective regulation of metabolic pathways in yeast. (a) Schematic diagram of 3‐hydroxypropionate (3HP) biosynthetic pathway in yeast. Upon blue light triggered expression of the BauA and McrN pathway, it can divert the central metabolism from the β‐alanine as the precursor substrate to 3HP production. Growth profile (b) and 3HP production (c) of strain CEN.PK2‐1C with pRS425‐McrN‐BauA under YNBG medium and ScOptoQS(α)4PF with pRS425‐McrN‐BauA in YNBD with 6 h blue light illumination. Growth profile (d) and 3HP production (e) of strain ScOptoQS(α)4PF with pRS425‐McrN‐BauA under different light conditions. (f) Fed‐batch bioreactor study for 3HP production. The fed‐batch experiments were carried out as following: after pre‐cultivated in SC media at 30°C for 12 h in the dark, the engineered strain ScOptoQS(α)4PF with pRS425‐McrN‐BauA was exposed to 100 µmol·m^−^^2^·s^−^^1^ blue light for 12 h and returned to dark cultivation. Data were obtained from three biologically independent samples and are presented as mean ± standard deviation for panel b‐c‐d‐e.

Subsequently, we attempted to implement the OptoQS circuit for metabolic engineering applications. As shown in Figure 5a, we applied our OptoQS circuit for the regulation of 3HP biosynthesis in yeast. In particular, we designed the biosynthetic pathway for 3HP biosynthesis that comprises BauA [38] and McrN [39] to utilize β‐alanine as the precursor substrate for 3HP synthesis. We first transformed plasmid pRS425Gal1/10‐McrN‐BauA into strain ScOptoQS(α)4PF for 3HP production. For a fair comparison, we also transformed the same plasmid into strain CEN.PK2‐1C for 3HP production using galactose as the carbon source. As shown in **5b**, no obvious change in the yeast cell growth after light exposure was observed in the 3HP‐producing yeast strains when compared to CEN.PK2‐1C, although the subtle phototoxic effect was not considered under the current experimental conditions [40]. The optogenetic‐regulated systems produced 3HP upon 6 h of blue light induction, and the engineered strain Sc3HP‐Opto with α‐factor ‐mediated OptoQS circuit produced 0.76 g/L 3HP at 48 h cultivation (Figure 5c), with production remaining elevated even after light removal due to the sustained activation (“recording”) of the circuit. To further confirm that 3HP biosynthesis is regulated by the optogenetic circuit, we compared the 3HP levels under different illumination conditions. As shown in Figure 5e, 3HP production was substantially lower under dark conditions, indicating that blue‐light stimulation is required to activate the biosynthetic pathway. In addition, experiments under varying light exposure durations revealed that both 3HP production and cell growth were dependent on the duration of illumination (Figure 5d.e), whereas prolonged illumination for 18 h would reduce 3‐HP production.

To further evaluate the robustness and scalability of this system, we conducted bioreactor‐scale cultivation experiments using the ScOptoQS(α)4PF strain. Under the fed‐batch condition, 3HP production continued to increase even after blue‐light removal, eventually reached 4.97 g/L at 84 h (Figure 5f), demonstrating that the optogenetic control strategy remains effective beyond shake‐tube cultures. Notably, EGFP fluorescence measurements collected during and after the illumination phase confirmed that the system remained activated even after the cessation of blue‐light exposure, thereby validating the functionality of the recording module at the bioreactor scale.

As the 3HP titers can be substantially improved by extensive engineering of yeast metabolism [39], it will be possible to further incorporate metabolic engineering strategies to improve the supply of precursors and cofactors [39] and the CO_2_ fixation strategy [41] to increase the 3HP titer in our optogenetic system. Taken together, these results demonstrated that light input is both necessary and tunable for pathway activation, while the engineered circuit enables sustained signal propagation and metabolic output at the population level across different cultivation scales. Moreover, since the light intensities and treatment durations are other key factors in affecting the optogenetic systems, it will be interesting to further optimize the light intensities and durations for an optimal 3HP production in future studies.

## 3 Discussion

Dynamic regulation of metabolic pathways to decouple the growth phase and production phase represents an effective strategy to improve strain performance with better productivity. Chemical inducers are frequently used to control metabolic enzyme expressions in microbial hosts [42, 43, 44]. However, the cost of chemical inducers becomes an inevitable concern for the downstream processes. With the recent development of synthetic biology, autonomous expression systems without relying on chemical inducers have been developed [8, 45]. However, the precise timing and tuning of quorum‐sensing‐regulated gene expression require extensive trial‐and‐error studies for the optimal strain performance. By exploring the light‐switchable transcription module of CRY2/CIB1 cryptochromes from *A. thaliana* [13, 24] to control α‐factor expression, we successfully connected the light‐sensing module with the yeast GPCR signaling pathway as the output module. The resulting α‐factor‐mediated OptoQS circuit utilizes this diffusible surrogate messenger to transmit the recorded signals across cell populations. As inspired by previous studies using blue light to induce α‐factor for studying intercellular communication and other behaviors [10, 46, 47], our system mainly focuses on addressing the light‐shading effect during fermentation processes, that is, harnessing OptoQS designs to overcome the light‐penetration limits and to achieve stable population‐level activation under minimal illumination. This design enables the conversion of localized or transient light inputs into coordinated responses across the entire cell population, thereby addressing limitations associated with light penetration and spatial heterogeneity. In the present study, we primarily employed a single light intensity to establish and validate the functionality of the OptoQS system. However, varying light intensity and stimulation regimes (e.g., pulse duration, frequency, and timing) could provide additional degrees of control over gene expression dynamics and system tunability, which will be systematically explored in future work. Considering a number of red light and near‐infrared optoswitches for deep tissue optogenetics in mammals have been recently developed [48, 49, 50, 51], it will be possible to explore similar designs of red light and near‐infrared optogenetics in yeast to further mitigate the light‐shading effect under large‐scale industrial fermentation.

To mimic the existing natural feedback systems [26], a self‐responsive feedforward autoinduction loop by an additional *P_PRE_‐*controlled α‐factor expression cassette was introduced to improve the signal transmission process and to increase the signal output. The engineered strain ScOptoQS(α)4PF with an additional *P_PRM2_*‐*MFα4* to accelerate the α‐factor accumulation could substantially enhance the signal output over that of its parental strain by more than 1.5‐fold at 36 h (Figure 3b). In the future, isolating more dynamic and sensitive pheromone‐responsive elements that respond to a low α‐factor concentration might be explored to the sensitivity of the α‐factor mediated OptoQS circuit. In addition, promoter engineering by introducing multiple configurational arrangements of pheromone‐responsive *cis*‐elements can alter the responses of synthetic promoters to pheromones [36], which might reduce the required time of blue light exposure to turn on the Gal4 expression, and to improve the genetic circuits with a better signal recording capability. For instance, a fast‐acting positive feedback loop to allow a saturated α‐factor accumulation in a short period (where its synthesis rate significantly exceeds degradation) is expected to create a “switch‐like” genetic response with minimal light input. However, the lack of tunability of gene expressions in this case will require tuning individual enzyme expressions at the promoter levels with different transcriptional activities of *P_GAL_* promoters [52, 53] to further balance the metabolic fluxes of the multi‐gene pathway. Concurrently, this system is designed with an inherent “recording” function, enabling sustained activation effects to be achieved with only a brief pulse of blue‐light stimulation, which minimizes the requirement for continuous illumination, thereby mitigating potential risks associated with phototoxicity while simultaneously ensuring the effective regulation of gene expression and product biosynthesis.

In summary, our work represents one of the pioneering works to report optogenetic regulation of quorum‐sensing circuits in yeast. While previous studies have successfully combined optogenetic control with signaling amplification mechanisms, our work extends these efforts by incorporating a quorum‐sensing‐mediated signal propagation layer into the circuit design. By coupling the light‐switchable control of diffusible surrogate messengers for population‐wide signal propagation, transient input is recorded into a self‐sustaining positive feedback loop to maintain gene expression after light removal. Based on the capability to transmit the signal across the cell population, these engineered strains would have potential applications for industrial‐scale and even solid‐phase fermentation. In addition, the OptoQS circuits are easy to adjust gene expression variability by merely modulating light conditions, which will be also an interesting feature for metabolic engineering purposes. Taken together, we envision that the design principles adopted here would be expanded to other microorganisms and eukaryotic cells, such as mammalian cell lines, for generating advanced genetic circuits.

## 4 Methods

### 4.1 Strains, Culture Media, and Reagents

*E. coli* TOP10 was used to clone plasmids. *S. cerevisiae* CEN.PK2‐1C from EUROSCARF was used as the parental strain for all yeast strain constructions. Biological reagents, including High‐Fidelity Phusion polymerase, T4 DNA ligase, and all restriction enzymes, were purchased from New England Biolabs (Beverly, MA, USA). Commercial kits for PCR purification, gel extraction, and plasmid DNA extraction were obtained from Sangon (Shanghai, China). All chemicals were purchased from Sigma–Aldrich or otherwise stated.

Luria‐Bertani (LB) medium (10 g/L tryptone, 5 g/L yeast extract, and 10 g/L NaCl) was used for cultivating the *E. coli* cells with the corresponding antibiotics. The antibiotic concentrations were ampicillin 100 µg/mL. Unless otherwise specified, yeast cells were grown at 30 °C on either YPD medium (10 g/L yeast extract, 20 g/L peptone and 20 g/L glucose) for culturing the parental yeast strain and engineered strains without plasmids or synthetic complete (SC) medium (20 g/L glucose, 6.7 g/L yeast nitrogen base without amino acids, 1.4 g/L yeast synthetic drop‐out medium supplements without histidine, leucine, tryptophan, and uracil) with appropriate dropouts (20 mg/L uracil, 100 mg/L leucine, 20 mg/L histidine, and 20 mg/mL tryptophan) for maintaining engineered strains with different auxotrophic selection markers. 1 g/L 5‐fluoroorotic acid (5‐FOA; Sigma–Aldrich) was used for recycling the gRNA‐expressing plasmids with the *URA3* marker. Next, 20 g/L agar (Sangon) was added to prepare solid media.

### 4.2 Plasmid and Strain Construction

Oligonucleotides used for plasmid constructions are listed in Table S1. Either the DNA assembler method or the Golden Gate assembly cloning kit (New England Biolabs) was used for plasmid construction, and all plasmids used are listed in Table S2. To achieve markerless genetic manipulation of yeast chromosomes, we used the CRISPR/Cas9‐mediated genome engineering approach as previously reported [42]. For the *FAR1* disruption, a PCR assembled fragment with 39 bp homologous regions to the *FAR1* locus was cotransformed with pgFar1 plasmid into the yeast competent cells harboring p414‐TEF1p‐Cas9, and the transformed cells were plated on SC‐Ura‐Trp for selection. The positive disruption of the *FAR1* gene was further verified by diagnostic PCR analysis. After removal of gRNA‐expressing plasmid by 5‐FOA treatment, successive rounds of genetic modifications were implemented in a similar way, to further yield *∆gal80*, *P_FUS1_‐GAL4*, *P_PRM2_‐Gal4*, *∆aro10::VP64‐CIB1*, *∆adh6::TetR‐CRY2*, *∆bar1::P_tetO7_*‐*MFα4*, *∆bar1::P_tetO7_*‐*MFα1*, *∆gal7‐10‐1::P_GAL1_*‐*EGFP*, *P_TEF2_‐GPA1*, *∆prm2::MFα4*, *∆ste2::Ptp2*, *∆sln1::AtCRE1*. The detailed flowchart for strain modifications is provided in Figure S5. In particular, the DNA fragment of *VP64* was amplified from plasmid M‐NMn‐VP64 [54]. The DNA fragments of *TetR and tetO7* were amplified from pCM225 [55]. All genetic information of engineered strains can be found in Table S3. Representative genetic verification results for strain ScOptoQS(α)4PF are provided in Figure S6.

### 4.3 Characterization of Genetic Circuits Using Fluorescent Reporter Gene

Plasmid pRS425Gal1‐EGFP [19] containing the signal “receiver” module of the *EGFP* gene under *P_GAL1_* promoter was transformed into the recipient cells by the heat‐shock method. For the control group, we replaced the *GAL1* promoter in pRS425Gal1‐EGFP with the PRM2 promoter, and the resulting plasmid was designated as pRS425Prm2‐EGFP. For collecting the dose‐response to different concentrations of Sc α‐factor (Sc peptide: WHWLQLKPGQPMY, synthesized by GenScript), experiments were carried out as follows. 14 mL sterile tubes containing 2 mL SC‐Leu medium with 2% glucose were inoculated with fresh overnight cultures to an initial OD_600_ (the optical density at 600 nm) of 2.0. Different concentrations of α‐factor were then supplemented (0, 0.001, 0.01, 0.1, 1, 10, 100, 1000, 10 000 nm). 100 µL of cell culture was periodically taken for measuring OD_600_ by a microplate reader (BioTek, Synergy H1) to determine the cell density, and green fluorescence intensities were recorded with excitation/emission at 476/512 nm.

For the characterization of optoswitches in yeast, experiments were carried out as following. 14 mL sterile tubes containing 2 mL YPD medium with 2% glucose were inoculated with fresh overnight cultures to an initial OD_600_ of 0.1. The cell cultures were incubated at 30°C and 200 rpm for 6 h in the dark conditions. After 6 h of incubation, when the culture reached an OD_600_ of 1–1.5, cells harboring the optogenetic circuit were transferred to a rotary shaker incubator equipped with a blue LED panel to induce α‐factor expression. Blue light intensity was set to 100 µmol·m^−^^2^·s^−^^1^, and cultures were maintained at 30°C and 200 rpm, with light cycles controlled according to experimental requirements. And 100 µL of cell culture in YPD was collected by centrifugation at 3000 *g* and resuspended with an equal volume of water for the measurement of OD_600_ and fluorescence intensities.

Fluorescence microscopy analysis was carried out using an inverted fluorescence microscope (Olympus IX51). Flow cytometry analysis was carried out using a Fortessa X‐20 flow cytometer. Briefly, cells were assayed at a low flow rate until 10 000 total events were collected using BD DIVA software on a Fortessa X‐20 flow cytometer. Flow cytometry data were analyzed using FlowJo (http://www.flowjo.com). Flow cytometry data analysis started by gating out debris by examining scatter plots with FSC‐A as the *x*‐axis and SSC‐A as the *y*‐axis, and drawing a gate to exclude outliers. A singlet gate was then drawn by examining scatter plots with FSC‐A as the *x*‐axis and FSC‐H as the *y*‐axis, and drawing the gate to exclude points not on the diagonal of the main body of the data points. To define GFP‐positive cells, a threshold was established using the negative control population (cells under dark conditions without positive feedback). Specifically, the GFP‐positive gate was set such that 99% of the negative control cells were classified as GFP‐negative. This threshold was then consistently applied to all samples. Within the GFP‐positive population with feedback, cells were further classified into low and high expression states. The boundary between these two states was defined as the local minimum (valley) between the two peaks in the fluorescence intensity distribution under blue light in the presence of positive feedback.

Fluorescence and optical density (OD_600_) measurements were taken using a microplate reader (BioTek, Synergy H1). The excitation and emission wavelengths used for EGFP fluorescence measurements were 476 and 512 nm, respectively. To normalize fluorescence signals, a two‐step background correction procedure was applied. First, background fluorescence from the culture medium exposed to the same light conditions was subtracted from the raw fluorescence measurements. In parallel, the OD_600_ value of blank media was subtracted from the measured OD_600_ to correct for background absorbance. Then, to account for autofluorescence and potential light‐induced effects on cellular components, control strains lacking the EGFP construct but subjected to the same light conditions were measured. The resulting baseline fluorescence (normalized by OD_600_) was then subtracted from the corresponding EGFP/OD_600_ values of each sample. Thus, the corrected fluorescence values reflect EGFP‐specific signals normalized to cell density and adjusted for both media background and cellular autofluorescence under identical illumination conditions. Reported values were calculated per the following formula.

$${\left(\mathrm{FL}/\mathrm{OD}\right)}_{\mathrm{corrected}}=\left(\mathrm{FL}-FL_{\mathrm{blank}}\right)/\left(\mathrm{OD}-OD_{\mathrm{blank}}\right)$$

### 4.4 Method for Modeling OptoQS Systems

The OptoQS system can be described by the following reactions:

In order to simplify the model and make it closer to the real situation, we limited the generation time of signal molecules to the first 12 h (including 6 h of dark culture and 6 h of blue light stimulation) with a reaction constant k1. The reaction is as follows:

Null→α−factor

α‐factor induces the phosphorylation of ste12, while α‐factor is not consumed during this process, with a reaction constant k2. The reaction is as follows:

α−factor→Ste12_P+α−factor

Ste12_P induces the generation of Gal4, while Ste12_P is not consumed during this process, with a reaction constant k3. The reaction is as follows:

Ste12_P→Gal4+Ste12_P

Then, Gal4 induces the generation of EGFP, while Gal4 is not consumed during this process, with a reaction constant k4. The reaction is as follows:

Gal4→EGFP+Gal4

Considering the positive feedback capability of the system, phosphorylated Ste12 can simultaneously activate α‐factor generation for signal recording and amplification. Ste12_P induces the generation of α‐factor, while Ste12_P is not consumed during this process, with a reaction constant k5. The reaction is as follows:

Ste12_P→α−factor+Ste12_P

Finally, α‐factor, Ste12_P, Gal4 and EGFP degrade with reaction constants kd1, kd2, kd3, kd4 respectively. The reactions are as follows:

α−factor→Null

Ste12_P→Null

Gal4→Null

EGFP→Null

To simulate the dynamics of EGFP, we used ODEs to model the above reactions. The ODEs are given as follows:

$$\frac{d\left[\alpha -factor\right]}{dt}=k1-kd1\left[\alpha -factor\right]+k5\left[ste12\_P\right]$$

$$\frac{d\left[Ste12\_P\right]}{dt}=k2\left[\alpha -factor\right]-kd2\left[Ste12\_P\right]$$

$$\frac{d\left[Gal4\right]}{dt}=k3\left[Ste12\_P\right]-kd3\left[Gal4\right]$$

$$\frac{d\left[EGFP\right]}{dt}=k4\left[Gal4\right]-kd4\left[EGFP\right]$$

After fitting the experimental data, the values of the reaction constants, as shown in Table S4, were obtained.

### 4.5 Cultivation and Analytical Methods for 3HP Production

For the cultivation of 3HP yeast strains that transformed with plasmid pRS425Gal1/10‐McrN‐BauA, 2 mL cultures were started in 14 mL shake tube by inoculating an amount of pre‐culture that resulted in a final optical density of 0.1 at 600 nm (OD_600_). The strains were grown at 30°C with 200 rpm. orbital shaking in defined SC minimal medium or YPD medium with 20 g/L glucose. Samples were periodically taken to measure the cell densities and 3HP concentrations. To test the applicability of the OptoQS circuit to synthesize 3HP in a laboratory‐scale fermenter, a fed‐batch experiment was performed using a 5 litre bioreactor (Infors HT). The seed culture was cultivated in the dark for overnight. Next day, 100 mL seed culture was inoculated into the bioreactor with a starting volume of 0.9 litre SC medium with 4% glucose. The fermentation temperature was maintained at 30°C, the air flow was set at a constant 2 L/min, and the mix rate was auto‐adjusted to maintain 20% dissolved oxygen. After inoculation, the bioreactor was exposed to blue light (450 nm) with an intensity of 100 µmol·m^−^^2^·s^−^^1^ for 12 h. During the feeding stage, 20 g glucose was supplemented every 24 h till the fermentation finishes. Samples were collected at 0, 6, 12, 24, 30, 36, 48, 54, 60, 72, 78, 84 and 96 h for the measurements of OD_600_, fluorescence, glucose and product levels.

Concentrations of 3HP and glucose were determined by HPLC. Culture samples were centrifuged at 12 000 *g* for 10 min, and the supernatants were filtered through a 0.22 µm nitrocellulose membrane. The filtrates were analyzed using an HPLC system (Shimadzu LC‐20A, Shimadzu, Japan) equipped with an Aminex HPX‐87H organic acid analysis column (300 mm x 7.8 mm, 9 µm) (Bio‐Rad, Hercules, USA) maintained at 55°C. Detection was performed using a refractive index detector (RID‐20A, Shimadzu, Japan), with 0.5 mm H_2_SO_4_ as the mobile phase at a flow rate of 0.8 mL/min.

### 4.6 Quantification and Statistical Analysis

Data were analyzed using GraphPad Prism version 9.0.1 and Excel Office. Dose effect curves were fitted using log (agonist) vs. response—variable slope (four parameters) in GraphPad Prism 9.0.1. Statistical significance between fluorescent signals was determined using one‐way analysis of variance (ANOVA) in GraphPad Prism. All the statistical details of experiments can be found in the figure legends and results. Figures were generated by Adobe Illustrator 2020.

## Author Contributions

J.Y. conceived of the project and designed the experiments. C.F. constructed all the strains and collected the data. H.C. performed the simulation using ODEs. Y.W., J.W., and Y.C. assisted with the data collection. Y.Z. and J.G. help with the data interpretation. J.Y., C.F., and H.C. interpreted the data and wrote the manuscript.

## Conflicts of Interest

The authors declare no conflicts of interest.

## Supporting information




**Supporting File**: advs75865‐sup‐0001‐SuppMat.docx.

## Data Availability

The data that supports the findings of this study are available in the supplementary material of this article.
