# Supplementary material for: Engineered Optogenetic Circuits In Yeast with Self‐Sustained Outputs
Source: Adv Sci (Weinh). 2026 May 27:e75865. Online ahead of print. doi: 10.1002/advs.75865 (PMC13336072; doi:10.1002/advs.75865)
Supplement: Supplementary file 1 — Supporting File: advs75865‐sup‐0001‐SuppMat.docx. [file ADVS-9999-e75865-s001.docx]

**Supplementary information**

**Engineered optogenetic circuits in yeast with self-sustained outputs**

Cong Fan^1,#^, Haofeng Chen^1,#^, Yan Wang^1^, Junyi Wang^1^, Yueyang Chen^1^, Yang Zhang^1^, Jinting Guan^2^, Jifeng Yuan^1,3^*

### Affiliations

^1^ State Key Laboratory of Cellular Stress Biology, School of Life Sciences, Faculty of Medicine and Life Sciences, Xiamen University, Fujian 361102, China

^2^ Department of Automation, Xiamen University, Fujian 361102, China

^3^ School of Advanced Interdisciplinary Biomedical Sciences, Faculty of Medicine and Life Sciences, Xiamen University, Fujian 361102, China

^#^ These authors contributed equally to the work.

*Corresponding author

Correspondence to: [jfyuan@xmu.edu.cn](mailto:jfyuan@xmu.edu.cn)

ORCRID: 0000-0003-1874-190X

**Supplementary Table 1** Oligonucleotides for plasmid construction and genome editing

| **Name** | **Description** |
| --- | --- |
| gFar1_fwd | TTGGTCTCAGATGGCGTCACGATCTCCACTTGGGTTTTAGAGCTAGAAATAG |
| gGal80_fwd | TTGGTCTCAGATGAACAAGCTCCTTTAGACCCGGTTTTAGAGCTAGAAATAG |
| gGal4p_fwd | TTGGTCTCAGATGGCACGCCATCATTTTAAGAGGTTTTAGAGCTAGAAATAG |
| gAro10_fwd | TTGGTCTCAGATGTGAACTGAACGCCGCTTATGGTTTTAGAGCTAGAAATAG |
| gAdh6_fwd | TTGGTCTCAGATGCTAGGGCCCAAGTCAAACAGGTTTTAGAGCTAGAAATAG |
| gBar1_fwd | TTGCTCTCAGATGGGTTTCTGCAATAAGCGAAGGTTTTAGAGCTAGAAATAG |
| gGal1_fwd | TTGGTCTCAGATGAGCAACGGCACAAATGAATGGTTTTAGAGCTAGAAATAG |
| gGpa1p_fwd | TTGGTCTCAGATGACTCAGTTTGGTCTTAGAAAGTTTTAGAGCTAGAAATAG |
| gPrm2_fwd | TTGGTCTCAGATGGCCTACATTCCACAAAAGAGGTTTTAGAGCTAGAAATAG |
| SUP4_down_rev | TTGGTCTCAAAAGAGACATAAAAAACAAAAAAAG |
| Far1_Del_fwd | ATGAAGACACCAACAAGAGTTTCGTTTGAAAAAAAAATGGTAGGGATAACAGGGTAATG |
| Far1_Del_rev | CTAGAGGTTGGGAACTTCCAGGGTCTGATGAATTCTTTCCCATTACCCTGTTATCCCTAC |
| Gal80_Del_fwd | ATGGACTACAACAAGAGATCTTCGGTCTCAACCGTGCCTACATTAAACG |
| Gal80_Del_rev | TTATAAACTATAATGCGAGATATTGCTAACGTTTAATGTAGGCACGGTTG |
| FUS1p_Gal4_fwd | TCTACAAACCTTCTATAATTTCAAAGTATTTACATAATTGAACTCCAATGGACAGCACG |
| FUS1p_Gal4_rev | AATATCGCATGCTTGTTCGATAGAAGACAGTAGCTTCATTTTGATTTTCAGAAACTT |
| PRM2p_Gal4_fwd | TCTACAAACCTTCTATAATTTCAAAGTATTTACATAATTTAGAAACACCAACAGATACG |
| PRM2p_Gal4_rev | AATATCGCATGCTTGTTCGATAGAAGACAGTAGCTTCATCTTTGCCGTACTCTTAG |
| Gal4_up | CGTGAGCGGCAAGAAGTTTC |
| Gal4in_down | GTTCTTCAGACACTTGGCGC |
| Aro10:VP64_fwd | ACTTCTGTAAAGTTTATTTACAAGATAACAAAGAAACAATGAGCAGGGCTGACCCCAAG |
| VP64_OE_rev | AGAGTTAATCAGCATGTCCAGGTCGAAATCATCAAGGGC |
| CIB1_OE_fwd | GCCCTTGATGATTTCGACCTGGACATGCTGATTAACTCTATGAATGGAGCTATAGGAGG |
| Aro10:CIB1_rev | TTGGTAGCAGTGTTTTATAATTGCGCCCACAAGTTTTCAAACTCCTAAATTGCCAT |
| Adh6:TetR_fwd | GAAGAAATTCAACACAACAACAAGAAAAGCCAAAATCATGTCTAGATTAGATAAAAGTA |
| TetR_OE_rev | AGACCCGTAATTGTTTTTCGTACGCGCGCGGCTGTACGC |
| CRY2_OE_fwd | GTACAGCCGCGCGCGTACGAAAAACAATTACGGGTCTATGAAGATGGACAAAAAGAC |
| Adh6:CRY2_rev | AAAGAAAGGAGCTACATTTATCAAGAGCTTGACAACTCATTTGCAACCATTTTTTC |
| Bar1:tetO7_fwd | TGACCTGCCACCACCTTCCTCTTCTTCTTGGGAGACCCGTAATTGTTTTTCG |
| tetO7_MFα_rev | CGAATAAAACTGCAGTAAAAATTGAAGGAAATCTCATGGATCCCCCGAATTGATCCG |
| Bar1:MFα1_fwd | TACCAAAATAAAAAGAGTGTCTAGAAGGGTCATATAATGAGATTTCCTTCAATTTTTAC |
| Bar1:MFα1_rev | GATATTTATATGCTATAAAGAAATTGTACTCCAGATTTCTTAGTACATTGGTTGGCCG |
| Bar1:MFα4_rev | TGATGGCTGCATAATATTACTATTTTGTTTTATATATCCTCTTAGGATTCGATTCAC |
| Bar1_up | CGAGTGTCACATAATAGCG |
| Bar1_down | TCAAAATTGTGATGGCTGC |
| Gal1-10-7:Gal1_fwd | GAGAAGTTGTTCTGAACAAAGTAAAAAAAAGAAGTATACACGGATTAGAAGCCGCCGAG |
| Gal1-10-7:Cyc1_rev | TAGAAAAAATATGATATGAATGAATATTCCACTTTCTTTCTTCGAGCGTCCCAAAACC |
| Gal1p_up | CTATACTTTAACGTCAAGGAG |
| Gal7_down | ATGGAAAGGACCACTCTTAC |
| TEF2p_Gpa1_fwd | GAGAGCAGAAATTTTTTTGTTACATATTGTTTTCCTTAAATATGGGGCCGTATACTTAC |
| TEF2p_Gpa1_rev | TTCGTCTCCTATTGTTTGCGTACTCACTGTACACCCCATGTTTAGTTAATTATAGTTCG |
| TEF2p_up | CATCACCTTCTTTTCTGGTG |
| Gpa1_in_down | CACGTTGTTTCTCCAGCTGC |
| PRM2:MFα_fwd | ACACCACTTTTCGATAAAACTAAGAGTACGGCAAAGATGAGATTTCCTTCAATTTTTAC |
| PRM2:MFα1_rev | CGAGACCGCTCGTGGAAGGTGACGATAATACGATACTTAGTACATTGGTTGGCCAGG |
| PRM2:MFα4_rev | CGAGACCGCTCGTGGAAGGTGACGATAATACGATACTTAACTGTTGTTATCAGTCGGGC |
| PRM2_up | TATCCTACCAAAAAGGTGTG |
| PRM2_down | TTGATCTGTAGGTCAGTAG |

| Prm2p_SacI_fwd | AGAGAGAGAGCTCTATCCTACCAAAAAGGTGTG |
| --- | --- |
| Prm2p_BamHI_rev | GACGGATCCGCCGTACTCTTAGTTTTATC |

| McrN_P1_fwd | TTGGTCTCATGAAACAATGAGCGGAACAGGACGACTG |
| --- | --- |
| McrN_P1_rev | TTGGTCTCAATCTATCCGACCGATGCACTGCG |
| McrN_OE_fwd | TTGGTCTCATTTCACTGCAGGCCGGCAAC |
| McrN_OE_rev | TTGGTCTCAGAaACCAGCCTGCTGGCCCG |
| BauA_P2_fwd | TTGGTCTCAAACCAATGAATCAGCCGCTGAACGT |
| BauA_P2_rev | TTGGTCTCATCTTACGCAATGCCGTTCAGCG |

**Supplementary Table 2** List of plasmids used in the present study.

| **Name** | **Description** |
| --- | --- |
| p414-TEF1p-Cas9 [^1^](#_ENREF_1) | Plasmid harboring the *Cas9* expression cassette |
| p426SNR52 | pESC-URA derivative with removal of *Bsa*I site in the *bla* selection marker and P_SNR52_-T_SUP4_ cassette |
| pRS425Gal1-EGFP [^2^](#_ENREF_2) | pRS425Gal1 derivative with P_GAL1_-*EGFP*-T_CYC1_ |
| pRS425Prm2-EGFP | pRS425Prm2 derivative with PPRM2-EGFP-TCYC1 |
| pRS425Gal1/10-McrN-BauA | pRS425Gal1/10 derivative with PGAL10-McrN-TADH1 and PGAL1-BauA-TCYC1 |
| pgFar1 | p426SNR52 derivative with gRNA targeting at the open reading frame of Far1 |
| pgGal4p | p426SNR52 derivative with gRNA targeting at the promoter region of Gal4 |
| pgGal80 | p426SNR52 derivative with gRNA targeting at the open reading frame of Gal80 |
| pgAro10 | p426SNR52 derivative with gRNA targeting at the open reading frame of Aro10 |
| pgAdh6 | p426SNR52 derivative with gRNA targeting at the open reading frame of Adh6 |
| pgBar1 | p426SNR52 derivative with gRNA targeting at the open reading frame of Bar1 |
| pgGal1 | p426SNR52 derivative with gRNA targeting at the open reading frame of Gal1 |
| pgGpa1p  pgPrm2 | p426SNR52 derivative with gRNA targeting at the promoter region of Gpa1  p426SNR52 derivative with gRNA targeting at the open reading frame of Prm2 |

**Supplementary Table 3** List of engineered strains used in the present study

| **Name** | **Description** |
| --- | --- |
| CEN.PK2-1C | MATa; his3D1; leu2-3_112; ura3-52; trp1-289; MAL2-8c; SUC2 |
| JY-Δfar1 | Strain CEN.PK2-1C derivative with Δ*far1* |
| JY-Δfar1Δgal80 | Strain CEN.PK2-1C derivative with Δ*far1* Δ*gal80* |
| ScG1 | Strain CEN.PK2-1C derivative with *∆far1 ∆gal80 P_FUS1_-Gal4* |
| ScG2 | Strain CEN.PK2-1C derivative with *∆far1 ∆gal80 P_PRM2_-Gal4* |
| ScBase | Strain CEN.PK2-1C derivative with *∆far1 ∆gal80 P_PRM2_-Gal4 ∆aro10::VP64-CIB1 ∆adh6::TetR-CRY2* |
| ScOptoQS(α)1 | Strain CEN.PK2-1C derivative with *∆far1 ∆gal80 P_PRM2_-Gal4 ∆aro10::VP64-CIB1 ∆adh6::TetR-CRY2 ∆bar1::P_tetO7_-MFα4 ∆gal7-10-1::P_GAL1_-EGFP* |
| ScOptoQS(α)2 | Strain CEN.PK2-1C derivative with *∆far1 ∆gal80 P_PRM2_-Gal4 ∆aro10::VP64-CIB1 ∆adh6::TetR-CRY2 ∆bar1::P_tetO7_-MFα1 ∆gal7-10-1::P_GAL1_-EGFP* |
| ScOptoQS(α)3 | Strain CEN.PK2-1C derivative with *∆far1 ∆gal80 P_PRM2_-Gal4 ∆aro10::VP64-CIB1 ∆adh6::TetR-CRY2 ∆bar1::P_tetO7_-MFα4 P_TEF2_-Gpa1 ∆gal7-10-1::P_GAL1_-EGFP* |
| ScOptoQS(α)4 | Strain CEN.PK2-1C derivative with *∆far1 ∆gal80 P_PRM2_-Gal4 ∆aro10::VP64-CIB1 ∆adh6::TetR-CRY2 ∆bar1::P_tetO7_-MFα1 P_TEF2_-Gpa1 ∆gal7-10-1::P_GAL1_-EGFP* |
| ScOptoQS(α)4PF | Strain CEN.PK2-1C derivative with *∆far1 ∆gal80 P_PRM2_-Gal4 ∆aro10::VP64-CIB1 ∆adh6::TetR-CRY2 ∆bar1::P_tetO7_-MFα1 P_TEF2_-Gpa1 ∆prm2::MFα4 ∆gal7-10-1::P_GAL1_-EGFP* |
| Sc3HP-Control | Strain CEN.PK2-1C transformed with plasmid pRS425Gal1/10-McrN-McrC |
| Sc3HP-Opto | Strain ScOptoQS(α)4PF transformed with plasmid pRS425Gal1/10-McrN-BauA |

| Parameter | Value | Units | Definition |
| --- | --- | --- | --- |
| k1 | 0.92245251 | h^-1^ | α-factor generation rate |
| k2 | 1.07090752 | h^-1^ | ste12 phosphorylation rate |
| k3 | 0.92245515 | h^-1^ | Gal4 generation rate |
| k4 | 0.92271235 | h^-1^ | EGFP generation rate |
| kd1 | 1.0603656 | h^-1^ | α-factor degradation rate |
| kd2 | 0.98539534 | h^-1^ | Phosphorylated ste12 degradation rate |
| kd3 | 0.72767402 | h^-1^ | Gal4 degradation rate |
| kd4 | 1.07697334 | h^-1^ | EGFP degradation rate |
| k5 | 1.10918895 | h^-1^ | Positive feedback generates α factor rate |

**Supplementary Table 4** Values of the reaction constants for ODE simulation of α-factor-mediated OptoQS circuits

**Supplementary Figures**

**
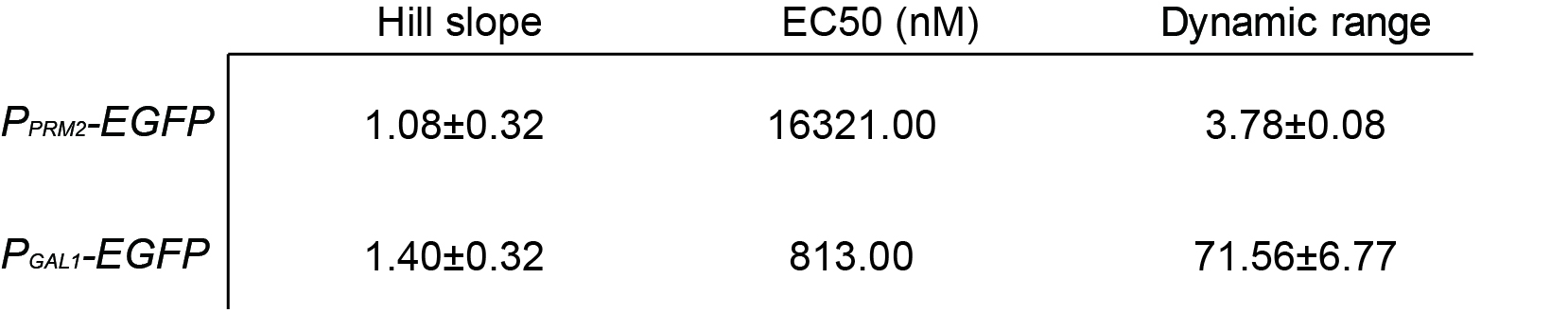
**

**Supplementary Fig. 1** Dynamic range, EC50 and Hillslope of ScG2 with a signal amplification module of *P_PRM2_-Gal4*. The control strain with *P_PRM2_*-*EGFP* is provided for a fair comparison. Data are obtained from three biologically independent samples and are presented as mean ± standard deviation.

**
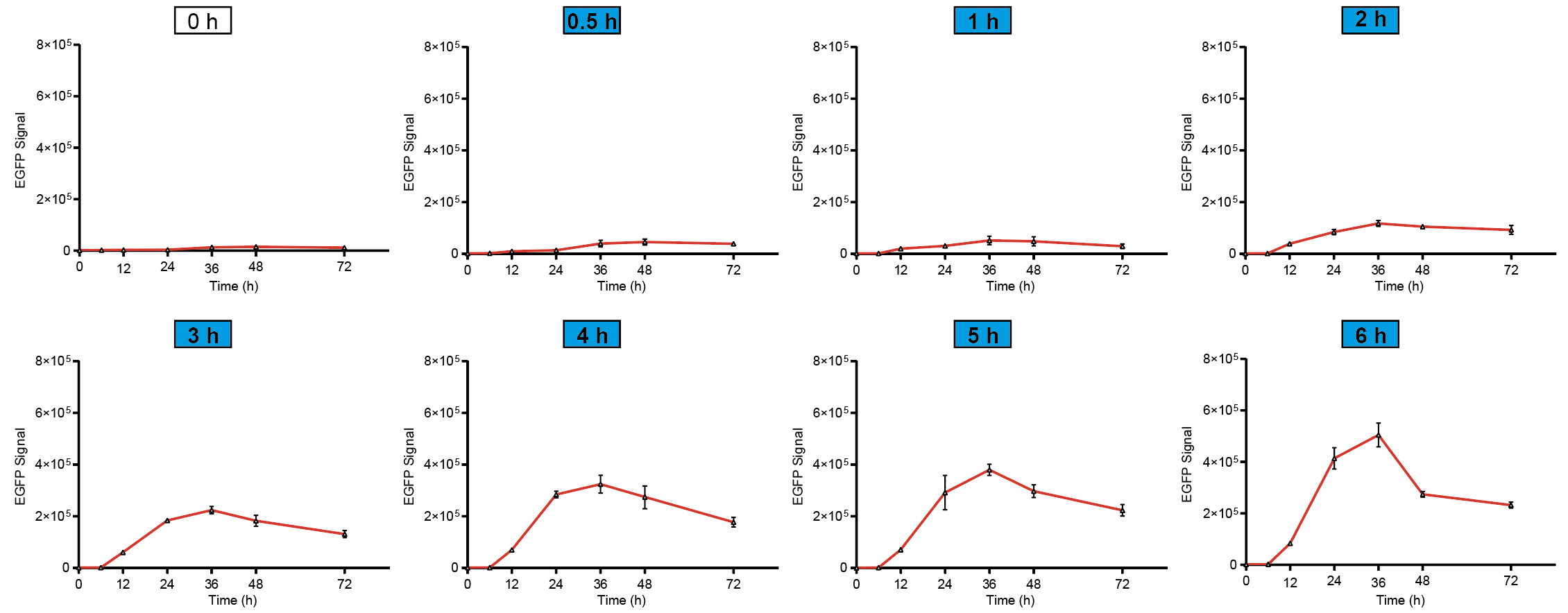
**

**Supplementary Fig. 2** Time course of EGFP signal outputs of the engineered strain ScOptoQS(α)4 under different durations of blue light exposure. All strains were first cultivated in YPD media at 30°C for 6 h in the dark. Subsequently, strains were exposed to 100 μmol·m⁻²·s⁻¹ blue light at different durations and returned to dark cultivation. Data are obtained from three biologically independent samples and are presented as mean ± standard deviation.

**Supplementary** **Fig. 3** Time course of EGFP signal outputs of the engineered strain ScG2 with the supernatant from ScOptoQS(α)4 yeast culture. Data are obtained from three biologically independent samples and are presented as mean ± standard deviation.


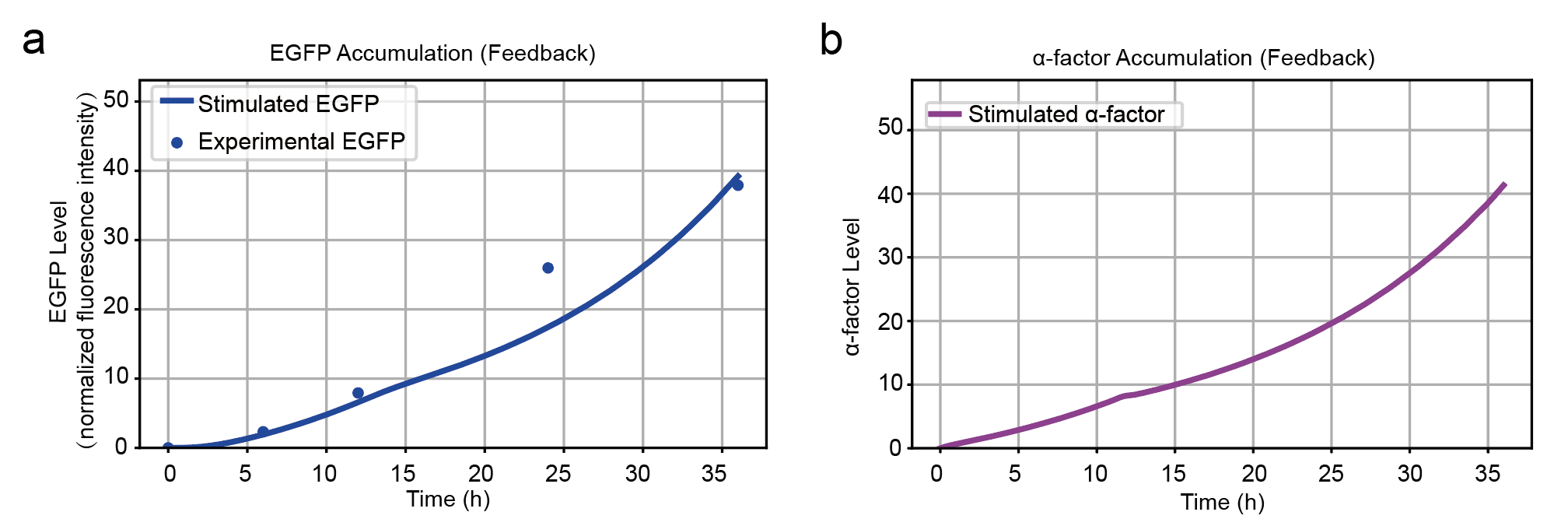


**Supplementary Fig. 4** Simulation of positive feedback circuits mediated by α-factor using ordinary differential equations (ODEs). The model predicts EGFP signal (**a**) and the accumulation levels of α-factor (**b**) in the positive feedback system after 6 h of light stimulation. All the parameters used in ODEs are provided in Supplementary Table 4.


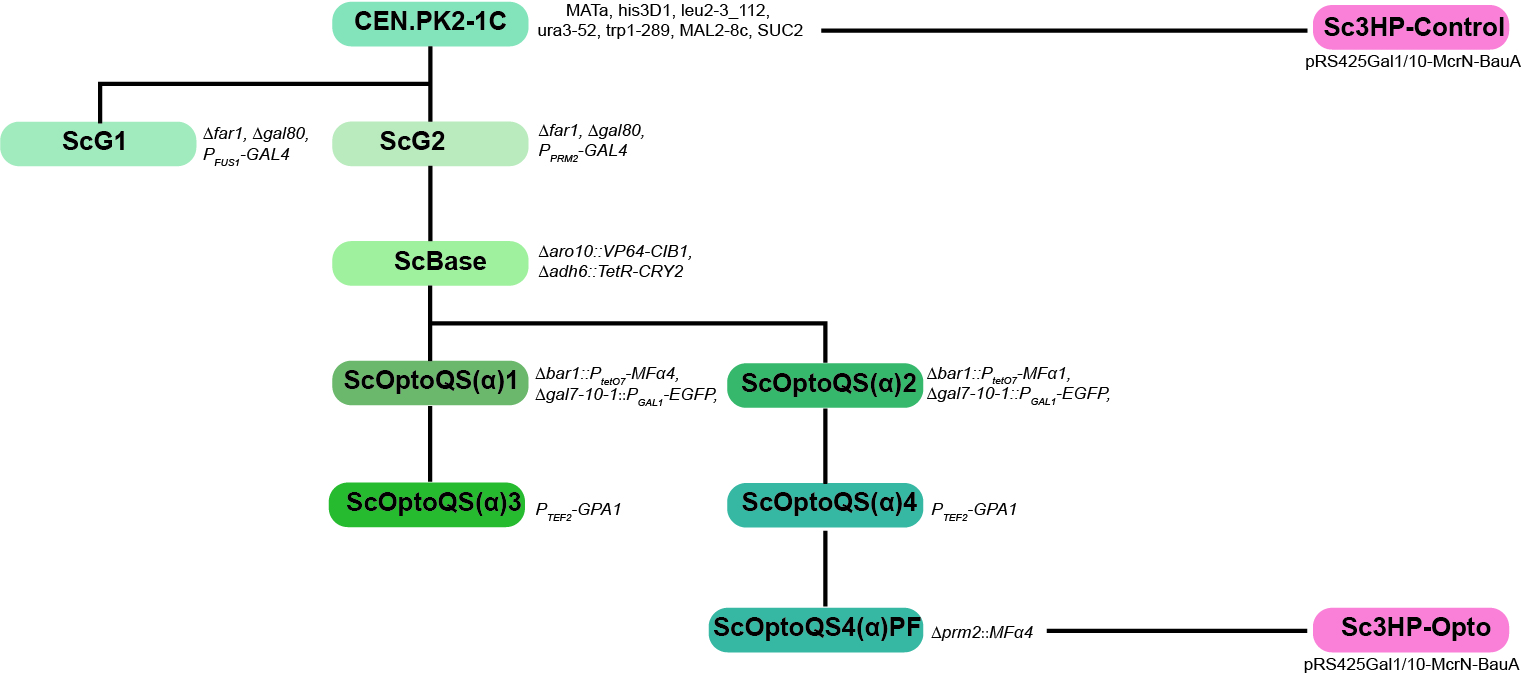


**Supplementary Fig. 5** Flow chart of yeast strain construction in this study.

**
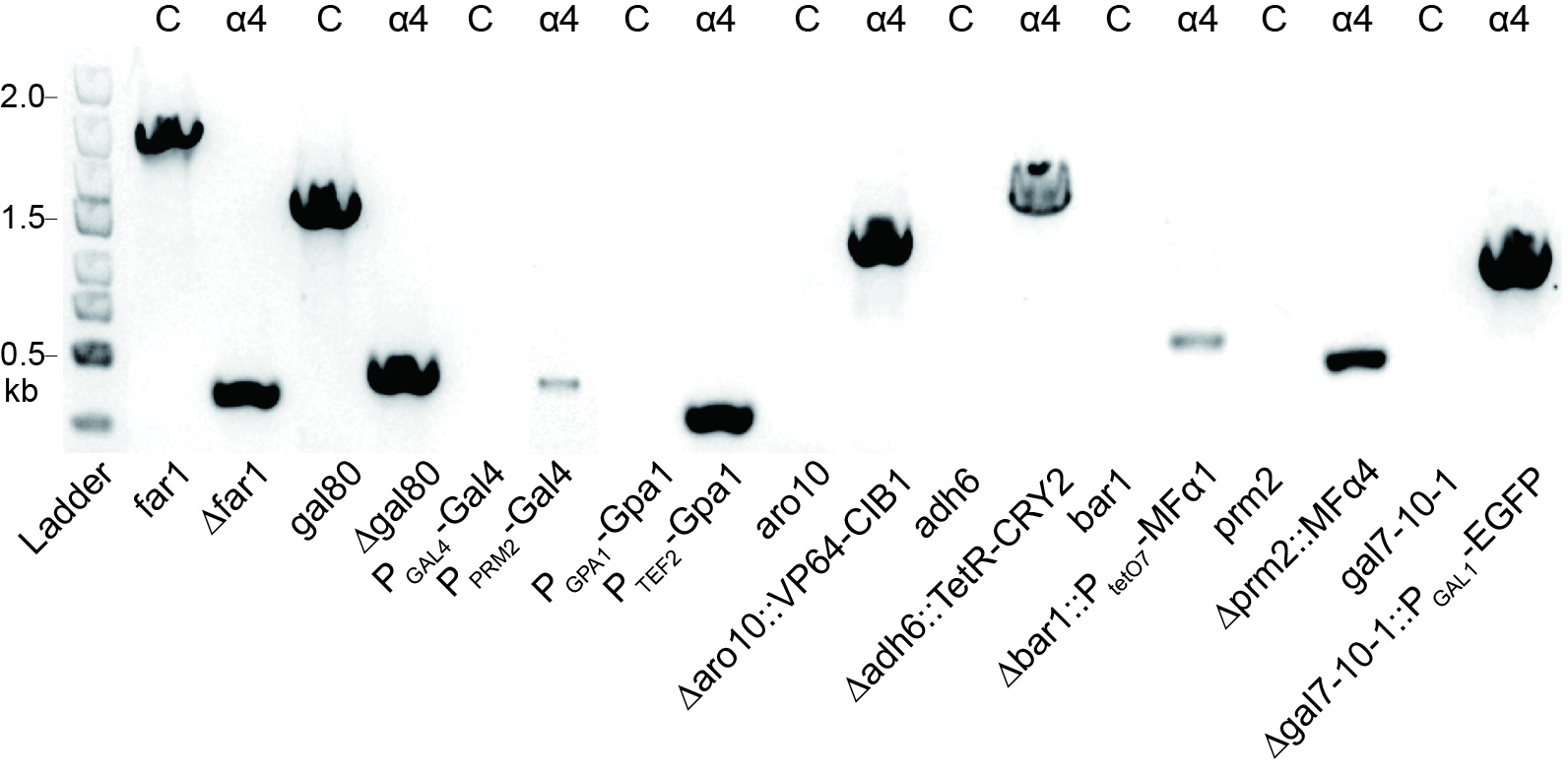
Supplementary Fig. 6** PCR verification of engineered strain ScOptoQS(α)4PF constructed by CRISPR-Cas9 mediated gene editing. C, control; α4 represents strain ScOptoQS(α)4PF.

**References**

1. DiCarlo, J.E., Norville, J.E., Mali, P., Rios, X., Aach, J., and Church, G.M. (2013). Genome engineering in Saccharomyces cerevisiae using CRISPR-Cas systems. *Nucleic Acids Res.* *41*, 4336-4343. <https://doi.org/10.1093/nar/gkt135>.

2. Fan, C., Zhang, D., Mo, Q., and Yuan, J. (2022). Engineering Saccharomyces cerevisiae-based biosensors for copper detection. *Microb. Biotechnol.* *15*, 2854-2860. [https://doi.org/10.1111/1751-7915.14105](https://doi.org/https://doi.org/10.1111/1751-7915.14105).
